# Co-producing a media campaign intervention to encourage Nigeria women’s engagement in early presentation to breast cancer screening: a qualitative study

**DOI:** 10.3389/fpubh.2026.1853334

**Published:** 2026-06-30

**Authors:** Tarela Juliet Ike, Dung Ezekiel Jidong, Evangelyn Ebi Ayobi, Mieyebi Lawrence Ike, Kelvin Odeyovwi Ayobi, Peremi Richmond Ike, Rukevwe Francis Doghor, Christopher Francis, Nusrat Husain

**Affiliations:** 1 Division of Psychology and Mental Health, University of Manchester, Manchester, United Kingdom; 2 School of Social Sciences, Humanities and Law, Teesside University, Middlesbrough, United Kingdom; 3 Division of Psychology and Mental Health, University of Manchester, Manchester, United Kingdom; 4 Manchester Global Foundation, London, United Kingdom; 5 Western Governors University, Salt Lake City, UT, United States; 6 Tare Wyd Legal Chambers, Warri, Nigeria; 7 Beryl Foundation, London, United Kingdom; 8 Nasarawa State University, Keffi, Nigeria; 9 Division of Psychology and Mental Health, University of Manchester, Merseycare NHS Foundation Trust, Manchester, United Kingdom

**Keywords:** breast cancer, co-production, early screening uptake, Nigeria, women

## Abstract

**Objective:**

Breast cancer significantly affects women globally, with women from low- and middle-income countries like Nigeria presenting late and at significant risk of mortality due to poor early screening. Yet a gap remains in the co-production of a media campaign intervention with women who are not or partially up to date with breast cancer screening using an interpretative phenomenological analytical lens.

**Method:**

Underpinned by a qualitative coproduction approach, a purposive sampling technique was adopted to recruit 11 participants, comprising women who are not or partially up to date with breast cancer from Delta state, who took part in a focus group discussion lasting approximately 90 min. The data was analysed using an interpretative phenomenological analysis lens.

**Finding:**

Based on the analyses the study identified the following five superordinate main themes, (i) Misinformation, fear, and the silencing effect of limited awareness, (ii) Inclusive awareness through multi-platform communication, (iii) Concise, compassionate messaging to motivate early screening, (iv) Empowering messages to counter myths and encourage action and (v), Authenticity underpinned by relatability in delivering persuasive health messages.

**Conclusion:**

This study offers valuable insights and original contributions to inform public health messaging policies globally on how culturally attuned, emotionally safe messaging can dismantle barriers and support earlier, more confident engagement with breast cancer screening. It also recommends the testing of the intervention to determine its effectiveness.

## Introduction

Breast cancer is a significant public health concern that mostly affects women, resulting in 670,000 deaths globally (1). Data from the World Health Organisation suggest that an estimated 2.3 million women are diagnosed with breast cancer globally (1). Of central concern are women from low- and middle-income countries like Nigeria who are at higher risk of presenting late to breast cancer screening and suffering an adverse mortality rate. This is further compounded by the prevalence rate in Nigeria, with an estimated incidence rate of 54.3–63 per 100,000 people and, annually, over 32,000 new cases diagnosed (2). In Nigeria, breast cancer also accounts for approximately 38.7% of all female cancers, with an estimated 88 women diagnosed daily, and often presenting at an advanced stage (2). A recent study also indicates that within the context of Africa, Nigeria reported the highest incidence (32,278) and 16,332 deaths emanating from breast cancer (3). Yet a gap remains in the co-production of a media campaign intervention with Nigerian women who are not up to date with breast cancer screening from an Interpretative Phenomenological Analytical (IPA) lens—constituting the key rationale for the present study. This study specifically addresses this gap using IPA to coproduce a media campaign intervention that addresses the behavioural and communication barriers to early screening uptake.

A growing body of literature has attempted to address the factors behind poor breast cancer screening uptake in Nigeria. Several studies argue that breast cancer screening remains critically low due to a convergence of educational (4), sociocultural barriers (5), financial constraints, and awareness-related barriers (6). Ehi et al. (7) study, drawing on data from 386 women in Kebbi State, Nigeria, indicated that an estimated 80.3% had never undergone a mammography screening, and the study also found that 66.3% of the respondents had never undergone a clinical breast examination. These deficits in breast cancer screening persist even among female undergraduates and postgraduate students, where it was found that although most acknowledged the importance of early detection, their understanding of screening procedures and diagnostic pathways remained inadequate, underscoring persistent screening gaps with an alarmingly low rate on only 17.5% of 548 survey respondents having undergone clinical breast examination (8). Another strand of literature further highlights the role of sociocultural norms in further exacerbating these knowledge-based barriers. Evidence from a study conducted in Abuja, Nigeria, indicated that sociocultural practices, spousal decision-making, and religious restrictions significantly impede women’s ability to seek screening services (31). These findings align with broader research indicating that stigma (9), cultural beliefs (4), and fear of diagnosis (10) remain potent deterrents to proactive health-seeking behaviour.

Other studies contend that health-system barriers (11, 12), economic challenges (13), and geographical constraints (4) compound these educational and sociocultural limitations. Expensive health care also appears to remain one of the most formidable obstacles, with surveyed respondents in Abuja citing financial constraints associated with the high cost of screening as a primary reason for avoiding screening—reflecting Nigeria’s heavy reliance on out-of-pocket health expenditures (14). Geographic inequity further intensifies exclusion, as diagnostic and imaging facilities are disproportionately concentrated in urban centres; national analyses highlight that access to essential screening infrastructure remains limited and uneven across regions, particularly disadvantaging rural women (15). At the health-system level, Nigeria also appears to lack a coordinated national breast cancer screening programme, and deficiencies in referral pathways, training, and diagnostic availability create additional obstacles. Collectively, these systemic failures—combined with entrenched cultural norms, financial hardship, and widespread misinformation arguably create a multidimensional barrier structure that continues to undermine efforts to improve early detection and reduce breast cancer mortality in Nigeria.

Yet a gap remains which the present study seeks to address using interpretative phenomenological analysis in the co-production process to foster awareness of the need for early breast cancer screening. The gap also emanates from an early phase of stakeholder and community engagement, during which participants underscored the need for a media-based intervention. This is also highlighted in our previous empirical research with Nigerian women not up to date with breast cancer screening, who identified the need for a targeted co-produced media campaign by and for them. As such, more specifically, IPA offers an original and significant lens for exploring the lived experiences of Nigerian women who are not or partially up to date with breast cancer screening, making it especially valuable for informing the co-creation of a media campaign intervention to encourage early screening. By centring women’s subjective meanings, sociocultural realities and lived experiences, IPA allows for the uncovering of why screening delays persist. This depth of insight is crucial in a context where, as indicated in previous literature barriers stem from complex intersections of knowledge gaps, fear, systemic constraints and cultural norms, IPA’s idiographic focus is also useful in that it ensures that the voices of women who are typically unheard—particularly those disengaged from preventive health practices—directly shape the development of the media campaign intervention.

In turn, a co-production approach is essential because it positions Black Nigerian women as knowledge-holders, ensuring the media intervention reflects their lived realities, cultural logics, and structural barriers rather than imposing externally constructed assumptions, ultimately strengthening both trust and uptake of screening. Using Interpretative Phenomenological Analysis (IPA) allows the project to surface the emotional, spiritual, and embodied meanings women attach to breast cancer, screening, and healthcare encounters, generating insights deep enough to shape messages that genuinely resonate rather than merely inform. Bringing co-production and IPA together creates an intervention that is culturally grounded, emotionally attuned, and structurally aware, increasing the likelihood that the campaign might potentially shift perceptions, reduce fear, foster behavioural change and motivate earlier screening among women who are partially or not up to date with screening in Nigeria. In essence, the primary research question this study seeks to address is:

- How can Nigerian women who are not or partially up to date with breast cancer screening inform the co-production of a media campaign to encourage early screening uptake?

To address the preceding research question, this study outlines the methodology employed, followed by a detailed presentation of the analysed findings. These findings are then discussed in relation to existing literature to highlight consistencies, divergences, and emerging insights. The chapter also examines the implications of these findings for policy development and for designing targeted interventions aimed at strengthening early breast cancer screening uptake. Through this approach, the study contributes evidence to support strategies that can reduce delayed presentation and ultimately improve survival outcomes.

## Methods

### Study design

The study adopts a case study design situated within an interpretivist epistemological stance (16) and a constructionist ontological position (17) to explore how Nigerian women who are not up to date with breast cancer screening can meaningfully shape the co-production of a culturally relevant media campaign intervention. An interpretivist approach enables a deep engagement with women lived experiences, beliefs, and contextual realities, recognising that understandings of breast cancer, health-seeking behaviour, and screening practices are socially and culturally mediated rather than fixed. Complementing this, a constructionist ontology acknowledges that knowledge about screening barriers and facilitators is co-constructed through interactions among participants, researchers, and the sociocultural environment. This design, including epistemological and ontological positioning underpinned by a qualitative method, allows the study to focus on depth rather than large numbers to capture the nuanced ways in which gender norms, health beliefs, stigma, and structural constraints shape women’s interpretations of screening, thereby generating insights that would be inaccessible through a positivist approach. By foregrounding women’s voices and experiences, the case study design provides a rich, contextualised foundation for collaboratively developing a media campaign that resonates with their realities, addresses their concerns, and enhances the relevance, acceptability, and effectiveness of interventions to improve breast cancer screening uptake.

### Ethics

The study applied for and granted ethical approval by the Jos University Teaching Hospital Health Research Ethics Committee in Nigeria with ethics approval reference number JUTH/DCS/IREC/127/XXXI/3295. The study also upheld the ethical standards outlined in the Declaration of Helsinki by ensuring informed, voluntary participation; providing clear information about the study’s purpose, procedures, risks, and benefits; and guaranteeing participants’ right to withdraw without being obliged to give a reason. Participants were also informed of confidentiality and anonymisation procedures to protect participants from stigma or unintended disclosure, particularly given the sensitivity surrounding women’s health and cancer-related fears. In addition, the participant was duly informed of how their data will be used, including for publications and presentations at conferences and seminars. All participants duly completed the electronic consent form prior to participation.

### Inclusion / exclusion criteria

The study’s inclusion criteria are that participants must be based in Nigeria, self-identify as female, be aged 18 + years, and be able to give informed consent before participating in the study. Participants must also be able to speak and understand English, the official language of Nigeria. To also meet the inclusion criteria, participant must not be up to date or partially up to date with their breast cancer screening. Regarding exclusion criteria, participants were excluded if they had severe mental health difficulties, were unable to provide informed consent, or did not meet the study inclusion criteria.

### Participant recruitment and demography

A purposive sampling technique was adopted to maximise recruitment. The rationale for adopting purposive sampling is that it is methodologically justified because it enables the intentional recruitment of women who are partially or not up to date with breast cancer screening, thereby ensuring that the data are generated from those whose lived experiences directly illuminate the determinants of delayed or non-uptake. This targeted strategy strengthens the analytic rigour and translational relevance of the study by capturing context-specific barriers and meaning-making processes essential to designing interventions aimed at improving screening participation in Nigeria. The participants were recruited from non-governmental organisations, local community centres, and faith-based organisations. We also leveraged snowball sampling, which is particularly effective for reaching Nigerian women who are not up to date with breast cancer screening, as it taps into trusted social networks, allowing initial participants to recruit others who share similar barriers and might otherwise remain inaccessible through conventional methods. By leveraging this interpersonal connection, especially in communities where sociocultural norms, fear, and limited awareness hinder screening engagement, the technique maximises recruitment depth and ensures richer access to the very women whose experiences are essential for the coproduction of an impactful intervention.

In total, 11 participants took part in the study. All 11 participants identified as female. The participant’s age range was from 18 to 60 years. The participant level of education includes *n* = 1 secondary; *n* = 10 indicated having attained tertiary-level education (including undergraduate and postgraduate degrees). For occupation, *n* = 8 indicated being employed, including self-employment, while *n* = 3 indicated being unemployed. The participants were recruited from Delta State, Nigeria; however, their state of origin spans across Nigeria, including Edo, Adamawa, Ondo, Osun, Delta, and Rivers states.

### Data collection

To ensure rigorous and contextually grounded data collection, the study established a dedicated steering committee comprising Nigerian women with lived experience of not being up to date or partially up to date with breast cancer screening. Their insights were instrumental in shaping the initial focus group questions, ensuring that the inquiry reflected the realities, priorities, and concerns of the population at the centre of the research. This process was further strengthened through an early phase of stakeholder and community engagement, during which participants underscored the need for a media-based intervention and provided detailed guidance on the types of questions required to ensure that such a campaign would authentically address community needs. Our previous empirical research with women not up to date with breast cancer screening also highlights the need for a media campaign intervention. The combined contributions of the steering committee, empirical research and community stakeholders feedback informed the development of a robust, culturally sensitive focus group guide, which was subsequently submitted for ethical review and received approval, thereby reinforcing the study’s methodological and ethical integrity.

One focus group discussion comprising 11 participants, lasting approximately 90 min was conducted. The rationale for the adoption of focus groups was due to its appropriateness for Interpretative Phenomenological Analysis as it create an interactive space where Nigerian women who are not or partially up to date with breast cancer screening can collectively explore, negotiate, and articulate their lived experiences, perceptions, and informational needs around breast-cancer screening—an essential component of IPA’s which as Palmer et al. (18) notes focus on shared meaning-making. The group setting also enabled the co-production of a media intervention, allowing participants to collaboratively shape content that reflects their cultural realities, barriers, and motivations rather than relying on researcher-driven assumptions. This aligns with co-production principles, which emphasise partnership and shared decision-making between researchers and communities to ensure interventions are more relevant, acceptable, and impactful (19).

Against the preceding backdrop, the focus group explored participants’ experiences with the factors limiting poor early breast cancer screening uptake and how to coproduce a media campaign intervention to encourage early screening uptake. The focus group was conducted by a member of the steering committee with lived experienced of not having had a breast cancer screening who was trained by the lead researcher with support from the lead researcher and a public health expert from the research team. Examples of questions asked include: What are your experiences or thoughts about breast cancer screening?” What factors do you think we need to consider when designing the media intervention to increase its likelihood of improving help-seeking and early screening uptake? What do you think should be the content of the media campaign intervention? Which key stakeholders do you think need to be involved in making the media campaign a success? What length do you think the media campaign should be, and why? What medium do you suggest should be targeted for dissemination (e.g., radio, TV, social media, etc.)? Which social media platform and why? Who do you think should deliver the media message in the media campaign and why? What do you think would encourage women to engage in and listen to the media campaign intervention? Are there any thoughts or suggestions you might want to share that might not have been covered?

### Data analysis

The data analysis followed a rigorous Interpretative Phenomenological Analysis (IPA) approach as enunciated by Eatough and Smith (20) to explore how women who are not up to date with breast cancer screening make sense of their experiences and how these meanings can inform the co-production of a media campaign intervention. In terms of operationalising the coproduction approach, prior to data analysis, four members of the steering committee comprising women who are not or partially up to date with breast cancer screening were trained in IPA analysis by the lead researcher to aid the co-production process alongside their direct involvement in the analysis.

The recorded focus group was first transcribed verbatim in preparation for analysis using the Nvivo software. The analysis process began with repeated, immersive readings of the focus group transcripts to ensure deep familiarity with participants’ narratives. Initial noting involved documenting descriptive, linguistic, and conceptual insights, which were systematically coded using a full coding approach to capture both explicit content and underlying interpretative meanings. Emergent themes were then developed by clustering related codes, allowing patterns, contradictions, and unique perspectives to surface. Through this iterative process, the analysis moved from individual-level meaning-making to shared experiential themes across participants. The interpretative layer of IPA enabled the research team to link personal experiences with broader sociocultural dynamics, providing a rich, nuanced understanding of how perceptions, fears, and structural barriers shape women’s engagement with breast cancer screening and their ideas for a culturally resonant media intervention. IPA has also been used in myriads of settings to explore lived experiences of victims affected by complex psychosocial needs (21–23).

To ensure analytic rigour and enhance the credibility of the findings, intercoder reliability procedures were embedded throughout the analysis. Three researchers comprising members of the steering group (women not or partially up to date with breast cancer screening) independently coded a subset of transcripts, comparing interpretations to identify convergence, divergence, and potential blind spots within the analytic process. Differences in coding were discussed in depth to ensure that thematic development was grounded in participants’ accounts rather than individual researcher assumptions. This collaborative reflexive dialogue strengthened transparency and supported the co-construction of themes aligned with IPA principles. The process also ensured that the themes informing the media campaign intervention were not only methodologically robust but also accurately rooted in women lived experiences. By integrating intercoder reliability into the IPA framework, the study upheld analytical consistency, reduced interpretative bias, and reinforced the legitimacy of the insights that shaped the co-produced media campaign aimed at improving early breast cancer screening uptake.

It is pertinent to add that the findings of the themes were used to inform the co-production of the brief media campaign intervention which was further validated by the steering committee members and five other participants who initially took part in the focus group discussion. The final message was named by the five other participants as “BrEast Cancer Screening AWAREness (BE-AWARE) campaign”, and the jingle script is contained in Appendix 1. In total, five superordinate themes emerged from the analysed data. Below is a table showing samples of the themes, along with supporting extracts (Table 1).

**Table 1:** Summary clustered superordinate themes and representative quotes.

Transcripts	Emergent themes	Clustered superordinate themes
“One thing I want to talk about is less awareness. People in the rural area may not know. They are not well-informed about the risk or what breast cancer actually is. So, these are my two points that I think may cause late attention to breast cancer screening.” (Morenikeji) “I always used to think breast cancer affects only older women who for example, smoke tobacco. So that sort of made me not bother to check, and I was not even aware it could affect younger women.” (Belema)	Poor awareness Uncertainty Risk misperception Underestimation of vulnerability	Misinformation, Fear, and the Silencing Effect of Limited Awareness
“I will say what should be in the body (of the media campaign) should be about how to differentiate between the lump of a cancer in the breast and something else, because some people hardly know how to differentiate. Someone I know is confused. She does not know what is happening in her breast, though. She’s still yet to go to the hospital because she is confused. So, I think the main body should also carry that.” (Gabriela) “I will like to add that they should also address the myths and fear that is associated with breast cancer, like they should try to make them understand that breast cancer is not caused by witchcraft or spiritual attack. And so, they should like, you know, they can present things like testimonials from survivors and some kind of expert interviews. I think that will do a whole lot of good.” (Sotonye)	Poor knowledge of breast cancer symptom Bodily uncertainty Fear, myths, confusion, and delay in help-seeking Empowerment through transformative health communication	Empowering Messages to Counter Myths and Encourage Action

### Findings

Based on analysis of the study data from an interpretative phenomenological analytical lens, the following main themes emerged, including (i), Misinformation, Fear, and the Silencing Effect of Limited Awareness, (ii) Inclusive Awareness Through Multi-Platform Communication, (iii) Concise, Compassionate Messaging to Motivate Early Screening, (iv) Empowering Messages to Counter Myths and Encourage Action and (v), Authenticity and Relatability in Delivering Persuasive Health Messages.

### Misinformation, fear, and the silencing effect of limited awareness

A dominant pattern across the participant accounts was how an incomplete or inaccurate understanding of breast cancer shapes their lived experience of risk, creating a sense of false security while simultaneously intensifying fear. In addition, limited awareness exacerbated by cultural narratives, rural isolation, and the absence of survivor stories appears to contribute to avoidance, delayed screening, and a personal struggle to make sense of conflicting meanings surrounding breast cancer. Talking about this, one participant said:

One thing I want to talk about is less awareness which also affected me. People in the rural area may not know. They are not well-informed about the risk or what breast cancer actually is. So, these are my two points that I think may cause late attention to breast cancer screening. (Morenikeji)

The participant’s account reveals a lived experience centred on *perceived informational deprivation* as a key barrier to early breast-cancer screening, highlighting the phenomenological sense of uncertainty and vulnerability that arises when individuals feel disconnected from essential health knowledge. By describing her poor awareness coupled with people in rural areas as “*not well-informed about the risk or what breast cancer actually is,”* the participant constructs a narrative of disadvantage rooted in structural and geographical inequity, suggesting that limited awareness is not simply an individual shortcoming but a contextual condition shaping how health is understood and acted upon. From a hermeneutic standpoint, the participant appears to be making sense of a broader socio-cultural landscape in which knowledge is unevenly distributed, implicitly interpreting rural settings as spaces of exclusion from mainstream health communication. Her language—particularly the repetition of *“not know”* and *“not well-informed”*—reflects an interpretive struggle to articulate how lack of awareness translates into delayed engagement with screening, thereby revealing her own meaning making process about risk and responsibility. The participant’s account also reveals the personal understanding of why late attention to breast cancer screening occurs, foregrounding her specific perspective on the interplay between environment and knowledge. Commenting on poor awareness, another participant also said:

I always used to think breast cancer affects only older women who for example, smoke or take tobacco. So that sort of made me not bother to check, and I was not even aware it could affect younger women. Then there is also the issue of feeling that being aware is tantamount to a death sentence, as I have hardly heard of any survivors, which also makes me afraid, coupled with the poor awareness (Belema)

The participant’s description reveals a deeply embedded set of beliefs about who is vulnerable to breast cancer, illustrating the phenomenological experience of navigating risk through culturally transmitted assumptions rather than medical knowledge. The participant’s initial conviction that breast cancer *“affects only older women who… smoke or tobacco”* demonstrates how personal understanding of illness is shaped by lay theories and social narratives, which in this case provided a false sense of immunity and reduced motivation to engage in screening. Hermeneutically, in making sense of the participant’s lived experience, the participant appears engaged in a complex meaning-making process, as she reinterprets her former assumptions in light of new awareness that younger women may also be affected. This shift reflects an internal dialogue between past understandings and present fears, particularly as the participant associates awareness with inevitability and death, noting that being informed feels like *“a death sentence.”* The phrase appears to signal an interpretation of breast cancer as universally fatal, underpinned by the absence of survivor stories within her community, which intensifies the participant’s fear and avoidance. Ideographically, this account highlights the participant’s unique biographical context, where limited exposure to survivor narratives, combined with poor public awareness, produces a personalised landscape of fear and misinformation. Focusing on this individual’s narrative illuminates how a specific history of misconception, emotional responses, and social environment converge to shape her health-seeking behaviour, offering insight into the personal and contextual barriers that contribute to delayed screening.

### Inclusive awareness through multi-platform communication

Across the extracts, participants emphasise the need for diverse and accessible communication channels ranging from highly engaging digital platforms like Facebook and TikTok to more traditional media such as radio and television—to effectively reach different groups of women. Their reflections highlight a clear recognition that breast cancer awareness must be tailored to varied technological realities and social contexts, ensuring that both online and offline communities are meaningfully included.

I feel Facebook is one social media platform that a good number of people are more active on. So, I feel that way like on Facebook, people are more likely to get educated. (Kambili)

In this extract, the participant positions Facebook as a meaningful and accessible space for public education, drawing on her own perception that *“a good number of people are more active on”* this platform. Phenomenologically, this reflects a lived understanding of social media not merely as entertainment but as a practical channel for health communication. The participant’s repeated use of *“I feel”* highlights a tentative yet personally grounded belief that engagement levels directly enhance opportunities for learning. From a hermeneutic perspective, the participant appears to interpret online activity as a proxy for potential impact, suggesting that visibility and familiarity are key drivers of health education. Her specific focus on Facebook—rather than social media more broadly—reveals a personalised lens shaped by her own experiences or observations, indicating that for this participant, effective health messaging is tightly linked to the platforms people already inhabit in their everyday lives. Another participant also said:

I think TikTok is one rapidly growing platform that can be used, especially among younger women, because of its entertaining short videos and strong engagement potential. So, I think TikTok will be another platform or a very good one. (Sotonye)

In the preceding extract, the participant’s reflection reveals a lived experience shaped by awareness of shifting digital cultures, in which platforms like TikTok are perceived as powerful tools for reaching younger women through engaging, entertaining content. Phenomenologically, the extract highlights the participant’s optimism and sense of possibility as she imagines social media as an accessible means of demystifying breast cancer information. In making sense of the lived experience, the participant appears to be reflecting on the relationship between attention, learning, and digital spaces, interpreting TikTok’s rapid growth and high engagement as indicators of its potential to bridge existing awareness gaps. This interpretation is rooted in the participant’s understanding of contemporary media trends, illustrating how her personal experiences with online platforms shape her view of effective health-education strategies. Together, the accounts offer rich insight into how the participants envision digital environments as transformative spaces for breast cancer awareness and prevention. While Facebook and TikTok were suggested, participants also recommended the need for dissemination via alternative platforms to aid inclusivity and reach. One participant said:

For the women in the rural community, I think we should have the radio, maybe a jingle on the radio and TV programmes, which can also be useful for those who are not online (Shola).

The participant’s account highlights a lived experience shaped by the awareness that many rural women remain excluded from digital health information, prompting the participant to view traditional media—such as radio jingles and television programmes—as vital alternatives. In making sense of the participants’ lived experiences, this reflects a sensitivity to the everyday realities of rural life, where limited internet access and technological barriers influence how breast cancer awareness is encountered and understood. From a hermeneutic perspective, the participant appears to be interpreting these media channels as culturally familiar and trusted modes of communication, suggesting that health messages delivered through them may carry greater credibility for those who are *“not online.”* The extract also reveals the participant’s personal understanding of rural information ecosystems, shaped by her own knowledge of how these communities typically engage with public messaging. The participant’s interpretation, therefore, appears to underscore a belief that awareness strategies must be adapted to local contexts, offering a nuanced view of how different communication modalities can support more equitable breast-cancer education.

### Concise, compassionate messaging to motivate early screening

Across the dataset, participants emphasise the importance of brief, emotionally sensitive messages that can capture attention while reducing the fear and stigma surrounding breast cancer screening. Their reflections highlight a belief that clear calls to action, normalising language, and accessible psychosocial support are essential for encouraging women to engage confidently in early screening.

For me, I feel it should be short. The message can be passed on in a short video, a jingle, or something similar. If it is long, I feel it may bore people, and they might not even engage with it. (Ivanta)

In this extract, the participant expresses a clear preference for concise health messaging, grounding this view is their lived sense that shorter content—such as *“a short video”* or “*a jingle”*—is more engaging and accessible. Phenomenologically, the participant’s concern centres on the risk of boredom and disengagement if messages are too long, reflecting an experiential awareness of how attention operates in everyday digital contexts. Her repeated use of *“I feel”* signals an interpretative process shaped by personal observations about how people typically respond to health information, revealing a belief that brevity fosters impact. In making sense of the participant account, her emphasis on short, creative formats illustrates a personalised understanding of effective communication, suggesting that meaningful health education appears to rely on immediacy, simplicity, and the ability to hold limited attention spans. Another participant also said:

Given my short attention span, a short message of 3-4 minutes is encouraged to aid retention and convey the key message swiftly. This is my view for both the radio jingle and the social media platforms, such as Facebook, YouTube, and TikTok. The message, while being brief, should also include a call to action so that women can go and check without feeling ashamed or afraid. It should also seek to address public stigma and encourage normalising of early screening and not stigmatise breast cancer, whilst providing psychosocial support channels for women who might need it to address the anxiety they face. That way, more women like myself will most likely engage in early screening (Belema)

The participant’s account indicates a lived experience shaped by the desire for concise, emotionally sensitive communication, reflecting an acute awareness of how attention, fear, and stigma intersect in shaping engagement with breast-cancer messages. Phenomenologically, her emphasis on a *“3–4-min”* message reveals an embodied understanding of how overwhelming or lengthy content can deter retention and amplify anxiety, particularly when the topic evokes fear, anxiety or perceived feeling of shame and stigma. The participant, therefore, appears to interpret both digital and traditional media as potential vehicles for empowerment, arguing that brief, action-oriented messages—combined with stigma reduction and psychosocial support—can reshape how women make sense of screening. The extract reflects the participant’s personal attentional limits and emotional perspective, which inform the belief that compassionate, non-judgemental communication will encourage earlier screening uptake. Overall, the participant’s interpretation reveals a nuanced understanding of how message tone, brevity, and emotional safety can influence women’s willingness to act.

### Empowering messages to counter myths and encourage action

Across the extracts, participants emphasise the need for breast-cancer communication that is clear, structured, and myth-breaking, ensuring women receive accurate guidance from introduction to call-to-action. Their reflections highlight the importance of combining practical information with survivor testimonials and expert reassurance to dismantle fear, challenge cultural misconceptions, and motivate early screening. Commenting on this, one participant said:

For me, I think the introduction should be maybe a brief information about breast cancer and why detecting early can make someone live longer or the benefit of detecting early. So, the main message is, OK, the main body could be how to self-examine how to detect yourself and the next is the need to break the stigma and also visiting the hospital, While the conclusion could be maybe the next step, where they can go get professional screening, who they can call and who they can speak to in case they want to speak up. (Shola).

The preceding extract highlights how the participants’ lived experience appears grounded in the desire for structured, reassuring health communication, revealing how clarity and ordered information help in navigating the emotional weight associated with breast cancer. The participant seeks an introduction that explains the life-extending value of early detection, suggesting that understanding *“why”* screening matters helps reduce uncertainty and fear. In making sense of her lived experiences, the participant constructs meaning through a step-by-step message sequence—from basic education to self-examination, stigma reduction, hospital visits, and access to professional support—interpreting this structure as essential for empowering women to act without shame. The extract highlights the participant’s own need for guidance, emotional safety, and direction, shaping her belief that women benefit from messages that clearly outline what breast cancer is, how to check, where to go, and who to contact. Overall, the participant’s meaning-making reveals a conviction that structured, compassionate messaging can seem to dismantle stigma and support women to pursue early screening confidently. Another participant also said:

I will like to add that they should also address the myths and fear that is associated with breast cancer, like they should try to make them understand that breast cancer is not caused by witchcraft or spiritual attack. And so, they should like, you know, they can present things like testimonials from survivors and some kind of expert interviews. I think that will do a whole lot of good. (Sotonye)

The participant’s reveals a lived experience that appears shaped by the desire to dismantle deeply rooted cultural myths and fears surrounding breast cancer, highlighting the emotional and social barriers that shape women’s engagement with screening. Phenomenologically, the participant recognises how beliefs about witchcraft and spiritual attack can generate fear and misunderstanding, prompting a need for messages that correct misconceptions while offering hope. In so doing, the participant interprets survivor testimonials and expert interviews as powerful tools for reshaping meaning, suggesting that hearing real stories and authoritative guidance can help women reinterpret breast cancer as manageable rather than fatalistic. In making sense of the participant’s account, the extract reflects the participant’s personal understanding of the stigma landscape within their community, shaping her belief that education must go beyond information to include emotional reassurance and culturally resonant myth-busting. Ultimately, the participant’s meaning-making underscores the conviction that culturally sensitive, evidence-based messaging can empower women and reduce the fear that deters early screening. Another participant, while commenting on the message content also said:

The conclusion should be that early detection doesn’t kill, and there's the empowerment message. Early detection does not kill and always go out there to check and that's alright. (Ufuoma)

In this extract, the participant frames early detection as a fundamentally empowering message, emphasising repeatedly that *“early detection does not kill,”* which reflects a lived effort to counter fear-based narratives surrounding breast cancer. This focus reveals the participant’s desire to replace anxiety with reassurance, positioning screening as an act of self-protection rather than danger. The participant’s assertive language *(“always go out there to check and that’s alright”*) conveys an interpretative stance that normalises health-seeking behaviour, suggesting that empowerment emerges through encouragement and permission to act. In essence, the participant appears to be making sense of how messaging can reshape individuals’ beliefs—challenging misconceptions and promoting agency. Her insistence on a clear, affirmative conclusion reveals a personal commitment to reframing screening as safe, positive, and achievable, highlighting what feels most meaningful to them: that knowledge and early action give individuals control rather than fear.

### Authenticity and relatability in delivering persuasive health messages

A notable pattern in participants’ lived experiences was their placing significant value on communication that feels authentic, relatable, and delivered by someone perceived as a credible voice. Their emphasis on tone, voice, and messenger appears to underscore the belief that how a message is conveyed is just as crucial as its content in motivating engagement and trust. Talking about this, one participant said:

Preferably a woman's voice because I feel like it is going to be more acceptable culturally for some women because it would be like, ok, this is a very sensitive issue that pertains to a woman, why is the man delivering the message? That is my take (Kambili).

The participant’s extract reflects a lived experience shaped by cultural expectations around gender and sensitivity, revealing a belief that messages about breast cancer are more credible and acceptable when voiced by another woman. Phenomenologically, this preference underscores how health communication is experienced not just as information but as an intimate exchange that feels more trustworthy when delivered by someone who shares the embodied reality of the issue. In making sense of the lived experience, the participant appears to be interpreting community norms, expressing an understanding that a male voice may provoke questions or discomfort, disrupting the intended message due to ingrained cultural assumptions about who should speak on women’s health. This insight emerges from the participant’s personal interpretation of social dynamics in her context, highlighting how her own cultural lens shapes what she perceives as appropriate and effective communication. The extract thus demonstrates how gendered expectations influence meaning-making and underscores the importance of culturally attuned messaging in promoting breast cancer awareness. Another participant also said:

For me, I will still say a woman voice should go for it […] and people will like to listen to more. (Fayosola).

In this extract, the participant expresses a personal conviction that a woman’s voice is the most appropriate and effective choice for delivering the message, grounding this view in the statement *“for me,”* which signals a deeply subjective and experiential judgement. This preference reflects the participant’s lived sense that certain voices carry greater credibility or resonance within the context of the topic, shaping how the message will be received. Through a hermeneutic lens, the participant interprets audience response by suggesting that people *“will like to listen to more,”* indicating an assumption that familiarity or perceived relatability enhances engagement. The focus on the specific characteristic of voice appears to reveal a uniquely personal framework for evaluating communication effectiveness, showing how individual beliefs and cultural understandings influence what the participant perceives as persuasive or trustworthy in health messaging.

## Discussion

The purpose of this study was to examine how Nigerian women who are not or partially up to date with breast cancer screening could inform the co-production of a media campaign to encourage early screening uptake. Informed by interpretative phenomenological analysis, the findings yield several superordinate themes which are discussed in relation to the literature alongside its policy implications.

First, the findings of this study reveal how women’s engagement with breast cancer awareness is shaped by a complex interplay of misinformation, cultural beliefs, fear, and communication preferences. Participants consistently highlighted limited awareness—particularly in rural communities—as a core barrier to early screening. This finding is congruent with evidence from previous literature, including in low and middle-income countries, which emphasises that early-detection inequities persist where awareness gaps and access barriers intersect, particularly in marginalised populations (24, 25). The participants’ expressions of uncertainty, fear, and reliance on lay beliefs mirror documented challenges in global breast cancer communication, where low health literacy and misinformation limit individuals’ ability to recognise symptoms and pursue timely care.

Secondly, another notable finding is the need for targeted media campaign intervention that addresses how cultural myths—such as beliefs attributing breast cancer to witchcraft or spiritual attack—build fear and stigma that deter screening. This aligns with previous literature showing that cultural assumptions (5), spiritual explanations, and misconceptions remain significant barriers to screening adherence among underserved women (26). Participants’ emphasis on myth-breaking messaging, survivor testimonials, and expert voices resonates with synthesised evidence on existing studies from the United States demonstrating that persuasive communication strategies, particularly those grounded in emotional appeals, narrative credibility, and cues to action, can meaningfully shift attitudes when culturally attuned and theoretically informed (26, 27).

Thirdly, digital media emerged as a central space through which participants believed breast-cancer awareness could be expanded—particularly via Facebook, YouTube, and TikTok, which they described as accessible, engaging, and widely used platforms. This is aligned with contemporary communication research showing that digital interventions (e.g., short videos, text-based reminders, and behavioural nudges) significantly increase screening uptake and reduce avoidance behaviours by meeting audiences in familiar online environments (28). At the same time, participants demonstrated sensitivity to the digital divide, particularly in rural communities where internet access is limited. Their insistence on radio and television as alternative channels corresponds with findings that multimodal and community-integrated communication strategies are essential to achieving equitable early-detection outcomes and care in low-resource contexts (29).

In addition, across the extracts, participants showed a strong preference for brief, concise messages, ideally 3 to 4 mins long, and structured in a way that reduces overwhelm. They articulated that shorter content improves retention and decreases fear—an insight that aligns directly with behavioural evidence showing that short, simple messages enhance uptake and comprehension of screening recommendations, especially among audiences with limited time or attention span. Their request for clear introductions, step-by-step guidance on self-examination, myth-busting segments, stigma-reduction content, and actionable conclusions reflects core components of effective early-detection education identified in public health literature (28).

Furthermore, our study also shows the importance of culturally sensitive delivery, strongly preferring that messages be voiced by women to foster trust, relatability, and emotional safety. This aligns with findings from communication and screening-equity studies, which identify gender-congruent messaging and culturally embedded communication as crucial to reducing discomfort, stigma, and mistrust, particularly in sensitive health contexts (26, 30). Moreover, the suggestion to use survivor stories aligns with previous literature, which stresses the persuasive impact of emotional appeals, narrative-based interventions, and testimonial framing in shaping health behaviours in breast cancer campaigns (26).

Collectively, these findings appear to validate the importance of multi-platform, concise, culturally attuned, and emotionally supportive messaging in addressing the intertwined barriers of stigma, misinformation, and limited awareness. They findings echo crucial calls for sustained, targeted, and context-responsive communication strategies as vital tools for advancing early detection and reducing inequities in breast cancer outcomes. Through the lens of IPA, these narratives illuminate how women make sense of breast cancer risk and awareness messaging within their socio-cultural environments, offering rich insights for designing interventions that resonate with lived experience and support earlier, more confident engagement with screening.

### Strength and limitations

A key limitation of this study is its reliance on a small, purposively selected sample, which limits the generalisability of the findings to broader populations. Additionally, the study was conducted in only one state in Nigeria and may not reflect experiences in other states. The study acknowledges the small sample size and its findings, which, while not generalizable, are not the core aims of qualitative research, which focuses on depth rather than large numbers, and still offer valuable insights that could be transferable to other contexts and countries sharing similar characteristics and the present study population. Another key limitation of this study is that the sample was predominantly composed of women with higher levels of education, which likely narrowed the range of perspectives captured. This educational skew means that the experiences and challenges of women with little or no formal education—who often face distinct socio-cultural, informational, and access-related barriers—are underrepresented. As a result, the findings may not adequately reflect the realities of more disadvantaged populations, particularly those in rural or underserved settings, where barriers to early breast cancer screening uptake are often more pronounced and context-specific.

To address the above limitations, future research is encouraged to prioritise the inclusion of a more diverse and representative sample by recruiting participants across different regions and educational backgrounds. Deliberate efforts should be made to engage women with limited or no formal education to ensure that the full spectrum of beliefs, constraints, and lived experiences influencing screening behaviours is captured. Such an approach will strengthen the generalisability of findings and better inform the design of equitable, culturally sensitive, and context-specific public health interventions aimed at improving early breast cancer screening uptake.

Furthermore, despite the limitations, the study’s strength lies in its contribution to knowledge through a co-production approach underpinned by Interpretative Phenomenological Analysis, which enabled an in-depth exploration of women’s lived experiences, cultural meanings, and emotional barriers to breast cancer screening, offering insights inaccessible through a quantitative approach. Its originality and significant contribution to practice also lie in co-producing the BE AWARE media-campaign intervention by and for women who were themselves due for screening—placing their voices at the centre of message design and ensuring that the content directly reflects their realities, concerns, and preferred communication formats. Thus, it illustrates its potential to raise awareness and promote early breast cancer screening uptake. The study’s significance is further demonstrated by how its findings align with evidence that culturally tailored, multi-platform, emotionally sensitive communication enhances early-detection engagement and reduces fear and misinformation in underserved groups. In addition, the methodological rigour represents another key strength of the study. Rigour was ensured through systematic IPA procedures, including iterative coding, detailed phenomenological engagement, reflexive interpretation, and triangulation with current literature on effective breast-cancer awareness messaging, which emphasises structured content, myth-busting strategies, survivor narratives, and clear calls to action as essential components of impactful health communication.

### Policy implication and recommendations

The present study holds significant policy implications as indicated in the need for policies that prioritise equitable, culturally tailored, and multi-platform breast-cancer awareness strategies, particularly for underserved and rural women who remain least informed yet most at risk. Public health policymakers are encouraged to implement sustained, targeted communication campaigns that incorporate brief, structured, myth-busting messages delivered through radio, television, and high-engagement digital platforms such as TikTok and Facebook, reflecting evidence that early-detection outcomes improve when awareness interventions are culturally resonant and accessible across communication divides. Based on the study’s findings, policies designed to foster effective media outreach are also encouraged to involve the inclusion of survivor testimonials, gender-appropriate messengers and expert guidance, aligning with research showing that communication barriers, cultural norms, and misinformation significantly shape screening behaviour and can be mitigated through supportive, trustworthy narratives (26). In addition, health systems are encouraged to embed these campaigns within broader early-detection frameworks that strengthen health literacy, reduce stigma, and equip communities with clear pathways to screening and follow-up care, complementing global recommendations for comprehensive, culturally sensitive early-detection programmes.

Finally, another policy implication and recommendation is the need to test the feasibility, acceptability, effectiveness and cost-effectiveness of the co-produced BrEast Cancer Screening AWAREness (BE-AWARE) campaign. This could be done using a randomised controlled trial design that compares the BE-AWARE campaign to existing public health interventions to provide robust evidence on its potential efficacy, with likely significant implications for fostering early screening uptake and reducing the health burden and care needs in Nigeria’s already overstretched health care system.

## Conclusion

Overall, this study demonstrates that women who are not up to date with breast cancer screening navigate a landscape shaped by limited awareness, cultural myths, stigma, fear and inconsistent access to trustworthy information—factors also identified internationally as barriers to early detection. Through the use of Interpretative Phenomenological Analysis and a co-production approach, the study illuminated how participants make sense of breast-cancer risk and identified their clear preferences for concise, culturally sensitive, emotionally supportive and multi-platform communication strategies, echoing wider evidence on the importance of targeted, audience-specific messaging for underserved groups. The BE-AWARE co-produced intervention —centred on myth-busting, clear self-examination guidance, accessible psychosocial support and gender-appropriate delivery—reflects both the lived realities of participants and best-practice recommendations for effective awareness campaigns in low-literacy and high-stigma settings. In conclusion, the study not only advances understanding of how women interpret and emotionally respond to breast cancer messaging but also provides an evidence-informed, culturally grounded input for developing communication interventions capable of motivating earlier and more confident screening uptake among women most at risk of late presentation.

## Data Availability

The original contributions presented in the study are included in the article/supplementary material, further inquiries can be directed to the corresponding author.

## Appendix 1

Co-produced jingle to encourage early breast cancer screening uptake.

**Table:**

Length	BrEast Cancer Screening AWAREness (BE-AWARE) campaign jingle to encourage early breast cancer screening uptake
3:00	My sister, as females, we carry hopes, joy, future and serve as primary caregivers or nurturers— but even the strongest woman needs care too! This care is especially crucial in sensitive region such as the breast which is a part of the body vulnerable to breast cancer. What is breast cancer: Breast cancer happens when abnormal cells in the breast grow uncontrollably, and while its exact cause is not fully known, factors such as age, family history, hormonal changes, and lifestyle can increase a woman’s risk. Breast cancer is not caused by witchcraft or spiritual factors— but early detection remains the most powerful protection. Breast cancer caught early is not a death sentence, and screening saves lives, yet many Nigerian women delay it because of fear, cost, or simply not knowing where to go. How can I check: Every woman can check her breasts monthly by standing or lying down, using the flat fingers of one hand to gently move in circles around the entire breast, the armpit, and the nipple, looking and feeling for any lumps, changes in shape, skin dimpling, or unusual discharge. What to do: My sister, your health is your strength: if you notice any change, or if you have not had a clinical breast screening in the past year, visit the nearest health centre for a simple, quick, and life-saving check-up. Screening is safe, affordable, and available in clinics near you, and going for your check-up does not mean something is wrong — it means you are choosing life. Your dreams matter, your children need you, your community needs you, and you deserve to live long, healthy, and strong. Survivors live healthy life due to early diagnosis and treatment. Do not wait and do not fear; go for your breast cancer screening today, because early detection saves lives — including yours. So, choose yourself today and go for your breast cancer screening at the nearest healthcare center or hospital and be aware!
